# Protein degradation rate is the dominant mechanism accounting for the differences in protein abundance of basal p53 in a human breast and colorectal cancer cell line

**DOI:** 10.1371/journal.pone.0177336

**Published:** 2017-05-10

**Authors:** Eszter Lakatos, Ali Salehi-Reyhani, Michael Barclay, Michael P. H. Stumpf, David R. Klug

**Affiliations:** 1 Department of Life Sciences, Centre for Integrative Systems Biology and Bioinformatics, Imperial College London, London, United Kingdom; 2 Department of Chemistry, Institute of Chemical Biology, Imperial College London, London, United Kingdom; Virginia Commonwealth University, UNITED STATES

## Abstract

We determine p53 protein abundances and cell to cell variation in two human cancer cell lines with single cell resolution, and show that the fractional width of the distributions is the same in both cases despite a large difference in average protein copy number. We developed a computational framework to identify dominant mechanisms controlling the variation of protein abundance in a simple model of gene expression from the summary statistics of single cell steady state protein expression distributions. Our results, based on single cell data analysed in a Bayesian framework, lends strong support to a model in which variation in the basal p53 protein abundance may be best explained by variations in the rate of p53 protein degradation. This is supported by measurements of the relative average levels of mRNA which are very similar despite large variation in the level of protein.

## Introduction

The regulation of gene expression and specifically the variation in protein abundance has received increasing attention. There are four main steps that contribute to protein abundance—transcription, mRNA degradation, translation and protein degradation.[1] The abundance of a specific protein is then determined by the relative rates of these steps and can vary by many orders of magnitude per cell. If the rate for one of these steps significantly differs to the others then it would be the dominant mechanism in determining the variation in protein abundance. There have been a number of reports including system-wide studies which conclude that the variation in protein abundance is predominantly controlled at either the level of transcription [2–4] or translation [5–8]. In the reports by Schwanhäusser et al. [7] and Li et al. [4], protein degradation only accounts for either 5% or 8%, respectively, of the variance in true protein levels.[9]

Our study here focuses on the major process that determines basal p53 protein abundance. p53 is not included in the studies by Schwanhäusser et al. [7] and Li et al. [4], therefore this paper represents the first analysis of whether the variation in basal p53 abundance is under degradation control. Mis-regulation of p53 has been shown to have a strong correlation with tumour development, with more than 50% of total cancer cases exhibiting a non-healthy p53 expression pattern.[10] In particular, the tumour suppressor protein is intrinsically involved in some specific cancers with over 96% of ovarian cancer cases involving some p53 mutation.[11]

One of the most important and widely investigated attributes of p53 is its relationship with MDM2, an E3 ubiquitin ligase, whose transcription is activated by p53. MDM2 readily binds to and ubiquitinates p53, targeting it for degradation by the proteasome.[12,13]The outcome of this relationship is that, under non-stressed conditions, p53 levels are maintained at a relatively low basal level; as any highly expressed p53 will readily promote the transcription of MDM2 and ultimately its own degradation.

On the other hand, in the case of stress response, this relationship can be interrupted in varying ways depending on the stress involved. For example, under conditions of DNA damage, induced by either ionizing radiation or ultraviolet irradiation, phosphorylation of both Ser15 of p53 [14–16] and MDM2 [17] prevents their interaction. The presence of activated oncogenes induces the ARF protein, which is able to bind and sequester MDM2.[18] Similarly, in the case of damage to the ribosome, large ribosomal RNAs leak into the nucleus and bind to MDM2 preventing its interaction with p53.[19,20] The outcome in both of these cases is the same: without its inhibitory binding partner, p53 is able to rapidly accumulate in cells and activate a downstream effector.

The relationship between p53 and MDM2 might be taken to imply that p53 protein is controlled predominately through degradation rather than repression of the gene itself. In order to investigate this we report, using single cell immunofluorescence measurements, how the steady-state distribution of p53 protein abundance in two human cancer cell lines may be analysed using a two-stage model of gene expression. With this model, we formalise the two competing hypotheses that either the rate of mRNA transcription or protein degradation is the predominant determinant of basal p53 protein abundance in these cells. Through subsequent Bayesian parameter estimation and model selection we conclude that differences in abundance levels within these cell types is predominantly determined by mechanisms which affect the rate of p53 protein degradation. This conclusion is perhaps expected in the light that that p53 expression in stressed conditions is orchestrated through protein degradation;[21] while on the other hand it provides a clear counterexample to the general finding that most protein levels are regulated by mRNA transcription.[4]

## Materials and methods

### Experimental methods

#### Cell culture and preparation

BE (gift from Institute of Cancer Research, UK), a human colon carcinoma cell line, and MCF7 (ATCC), a human breast adenocarcinoma cell line, cells were cultured using high glucose Dulbecco’s Modified Eagles Medium (DMEM; Invitrogen, UK) supplemented with 10% (v/v) foetal bovine serum (FBS; Sigma, UK) in polystyrene flasks in a 5% CO_2_ 37°C cell incubator.

Cells used in experiments were grown on 8 well chambered 25mm x 75mm slides (Nunc Lab-Tek II, Thermo Scientific). To improve cell adhesion, all chambers were fibronectin coated by treating each chamber with 200μL 50μg mL^-1^ fibronectin in phosphate buffered saline (PBS) for 30 minutes at room temperature. Fibronectin solution was aspirated and chambers were rinsed with PBS before seeding cells.

#### Immunostaining of fixed cells

Cells were fixed, permeabilised then immunostained following methods described in Stadler et al.[22] Only one cell type was seeded per well chamber. At ~80% confluence cells were removed from the incubator and washed 3× with ice cold PBS. For fixation, cells were treated with freshly made ice cold 4% paraformaldehyde in cell growth media then washed 3× with PBS. Cells were permeabilised using 0.1% (w/v) Triton X-100 for a total exposure of 15min, replacing the solution every 5min. Cells were incubated overnight at 4°C with 1μg mL^-1^ anti-p53 antibody (DO-1; Santa Cruz, Europe) labelled with Alexa Fluor 647 in 4% PBSA. After washing 4× with ice cold PBS for 10min, cell nuclei were stained with 300nM Hoechst 33342 in PBS and washed again 4× with ice cold PBS for 10min. Wells were filled with 78% glycerol in PBS, sealed and stored at 4°C before image acquisition.

#### Image acquisition and analysis

Wide-field fluorescence microscopy was performed on an inverted microscope (Nikon Ti-E, Nikon, Japan) using a mercury lamp (Nikon, Japan) using standard DAPI, FITC and Cy5 filter sets (Nikon, Japan). Images were acquired with an electron-multiplied CCD camera (IXON DU-897E; Andor Technologies, Ireland). The EM-CCD camera settings were as follows; 900ms acquisition time, electron multiplier gain 10 and the readout mode set to 1MHz at 16-bit. Tile scans of 4 x 4 fields of view (~0.5mm x 0.5 mm) were acquired for each fluorescence channel using a 60×, NA = 1.49 oil immersion objective. The focal plane was maintained by a Nikon Perfect Focus System (Nikon, Japan) and a motorised stage ensured good overlap between image channels. The microscope platform was automatically controlled using the NIS Elements software (Nikon, Japan) during acquisitions.

All image analysis to determine the single cell fluorescence intensity in each channel was automated using algorithms written for FiJi.[23] To compensate for the epifluorescence illumination profile of the microscope, tile scans of fluorophores in a mixed solution were acquired and used to flatten images.

#### qPCR RNA isolation

Total RNA was obtained from cultured cells using the commercially available MagMAX Total RNA Isolation Kit (LifeTechnologies, USA). Cells were cultured onto 6 well plates until they reached ~80% confluency. The media was removed and cells were lysed using the provided lysis buffer. The supernatants from each well were transferred to a 96 well plate. Magnetic beads were then added to bind all available RNA and the plate was placed on a magnetic stand (LifeTechnologies, USA). The bound RNA was washed multiple times using the provided wash solutions followed by the addition of TURBO DNAse to remove any DNA in the cell lysis solution. Once this was complete the RNA was rebound to the binding beads for a final wash step before being eluted into an elution buffer for storage. Total RNA yield was determined by performing an A260 measurement on a Nanodrop 2000 Spectrophotometer and was recorded for later use. RNA purity and quality was assessed using the ratio between A260 and A280 measurements and ensuring it fell within expected bounds (1.8–2.1). Isolated RNA was stored at -20°C in an RNAse free container until ready for use.

RNA was converted to cDNA through a reverse transcription reaction. Based on previously obtained RNA yields, RNA was diluted into 10μL diethylpyrocarbonate (DEPC) treated water to provide 2μg for the reverse transcription process. This was mixed with a 2X RT master mix solution containing the Reverse Transcriptase as well as the relevant buffer, dNTP mix and random primers. The total 20μL reaction volumes were prepared in individual Eppendorf vials and loaded onto a PCR thermocycler for the reaction itself. The vials were heated to 25°C for 10 minutes, then to 37°C for 120 minutes and 86°C for 5 minutes. The produced cDNA was stored at -20°C. The qPCR measurement was performed using a TAQMan gene expression assay for TP53 (Hs01034249_m1, LifeTechnologies, USA). For each cDNA sample this assay was in a real time PCR machine (ABI Prism 7700, Applied Biosystems, USA) and the cycle at which the TP53 amplification could be analysed (C_T_) was recorded and compared with that of the housekeeping gene HPRT1 (Hs02800695_m1, Life Technologies, USA). Relative mRNA levels were calculated using the comparative C_T_ method normalised to the first MCF7 sample.[24]

### Computational methods

#### Model formulation

**Fig 1:**
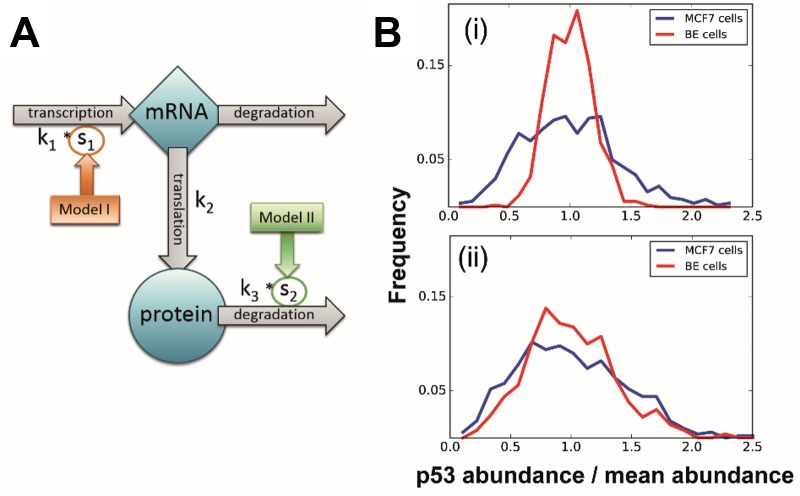
Schematics of the model and simulation examples of regulation strategies. **(A)** Schematic representation of the model of stochastic p53 protein expression. Blue shapes represent the two species, grey arrows the reactions modelled explicitly in our formalisation. Rate parameters corresponding to each reaction (*k*_1,_ *k*_2,_ *k*_3_) are indicated next to their respective reactions; the rate of mRNA degradation is assumed to be 1, and other rates are scaled accordingly. The two competing regulatory models are indicated in dark orange and green, with arrows pointing out the relevant reaction influenced through a scaling factor in each model. **(B)** Comparison of mRNA-level and protein-level regulation. Curves show p53 distribution in cell populations simulated using (i) the transcription-based and (ii) the protein degradation-based model. Blue and red distributions represent MCF7 and BE cell line populations, respectively. Protein abundance on the horizontal axis is normalised by mean p53 level to help comparison. Note the relative fractional widths in each case.

The underlying dynamics were modelled with a simple linear model of stochastic gene expression that consisted of two variables (mRNA and protein copy numbers) and four reactions: transcription, translation, and mRNA and protein degradation (Fig 1A). Regulatory processes and molecules influencing this system were implicitly modelled in the rate values of the individual reactions, which were inferred in the next step. To aid the analysis, the parameters were normalised by the mRNA degradation rate and we focused our study on the three remaining rate parameters of the process, thus obtaining the estimated rates in an arbitrary time-unit based on the mRNA half-life.

The two cell lines were assumed to both follow the simplified model with the same rate parameters, except for the regulatory reaction (see Fig 1A). The proposed mechanisms of controlling baseline expression (transcription- or protein degradation-based) were modelled in the form of a regulatory factor (*s*_1_ or *s*_2_), resulting in two models with either *s*_1_ or *s*_2_ accounting for the difference between cell lines (model I and model II, respectively).

#### Model comparison and inference

Model comparison and parameter estimation were carried out with an Approximate Bayesian Computation (ABC) method combined with sequential Monte Carlo (SMC) sampling, [25] implemented in the ABC-SysBio python package and complemented with the GPU-accelerated simulation tool, cudasim. [26,27] Each experiment made up of a pair of p53 distributions were analysed independently.

The model was simulated using a Stochastic Differential Equation (SDE) formulation and solved numerically with the Euler-Maruyama method.[28] For each parameter combination tested we simulated *n* instances of the system (*n* being the number of cells measured in the experimental data) and compared the simulated to the measured data set, in order to obtain parameters that have high *posterior* probability in light of the data. For the evaluation of the distance between simulated and target outputs, we used the Kolmogorov-Smirnov distance, computed as *d*_*KS*_ = *sup*_*x*∈ℝ_|*P*_1_(*X* ≤ *x*) − *P*_2_(*X* ≤ *x*) where *P*_1_ and *P*_2_ were the experimental and the simulated distribution, respectively. Distances were evaluated independently for each cell line sample, and the total distance was taken as their maximum. Incorporation of information on mRNA expression was done through an additive term in the total distance function, penalising significant differences between mRNA levels: $\frac{\left|avg(m_{1}\text{)}-avg(m_{2})\right|}{avg(m_{1})+avg(m_{2})}\mu$. The weight factor, *μ*, of the penalty term allowed for fine-tuning of the algorithm.

Model selection was carried out through an iterative process throughout which accepted parameters provided an increasingly better fit to the experimental data. The threshold values of the iterations were set to reject parameter combinations with a certain *α*_*i*_ significance, where *α*_*i*_ < *α*_*i*+1_ and the final threshold was *α*_*f*_ = 0.5. In each iteration, we sample enough parameters until we have 3000 accepted parameters. Models were evaluated based on the percentage of accepted samples obtained from each model, yielding a model evidence value between 0 and 1, with both models starting from 0.5 evidence. The algorithm was initialised with fixed initial conditions *protein*_*init*_ = 4 × 10^6^; *mrna*_*init*_ = 100 and uniform priors for all parameters. The ranges of uniform distributions are listed in S1 Table.

The algorithm was validated on synthetic datasets designed to reflect the characteristics of experimentally observed distribution, but with known rate parameters and henceforth known dominant regulatory mechanism. Details of synthetic data generation and results of the analysis are given in supplementary material (S2 Table and S4 Fig).

## Results and discussion

### Basal expression of p53

**Fig 2:**
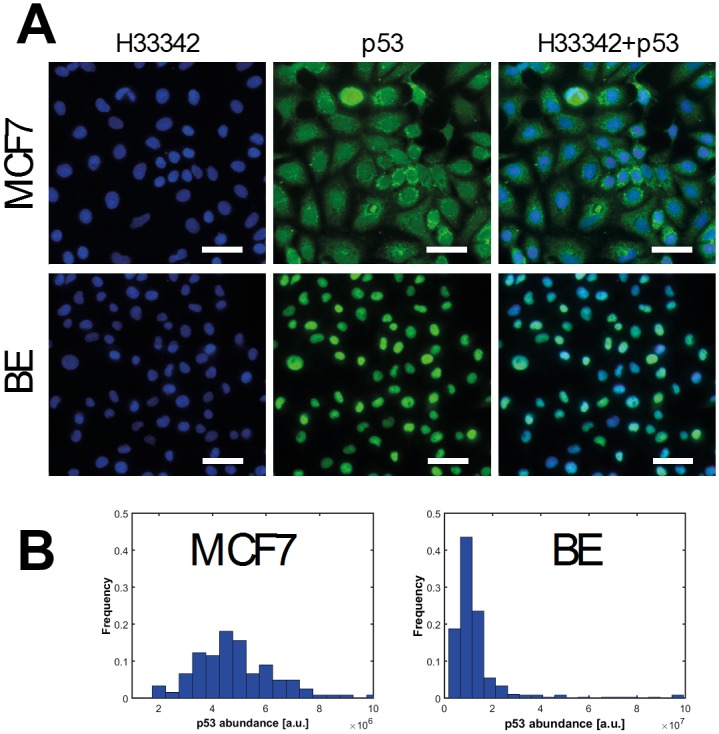
Results of single cell p53 immunofluorescence measurements. **(A)** Representative raw immunofluorescence images of fixed MCF7 (top row) and BE (bottom row) cells. Cells were stained directly using mouse monoclonal α-p53 (DO-1) conjugated to Alexa Fluor 488 to visualise p53 protein (shown in green) and counterstained with Hoechst 33342 to stain the nucleus (shown in blue). Overlaid images show that the p53 antibody staining pattern is both cytoplasmic and nuclear in MCF7 cells, whereas in BE cells only the nucleus is stained. Scale bars 50μm. **(B)** Immunofluorescence images are processed and the p53 fluorescence intensity per cell is determined. Distributions of p53 fluorescence total intensity per cell for MCF7 (n = 122) and BE (n = 400) cells. Note the scale of arbitrary fluorescence units.

Immunofluorescence experiments were conducted to determine the expression of p53 protein in MCF7 and BE cells. Individual cells were segmented in large field images so that analysis could be performed with single cell resolution. From the pattern of staining, p53 is localised in the nucleus in BE cells and at undetectable levels in the cytoplasm whereas in MCF7 cells p53 has detectable nuclear and cytoplasmic fractions (Fig 2A). As determined by the total fluorescence signal per cell, the mean basal expression of p53 protein is 2.6-fold higher in BE cells (mean ± SD, 1.27 ± 1.21 × 10^7^ a.u.) compared to MCF7 cells (4.91 ± 1.94 × 10^6^ a.u.). p53 expression spans a wide range (Fig 2B) and we have previously shown, albeit under different experimental conditions, that absolute p53 abundance in MCF7 and BE cell lines varies in the range of 10^4^−10^6^ p53 proteins per cell.[29,30]

MCF7 cells have wild-type p53 whereas the status of p53 in BE cells is unknown. Wild-type p53 is generally presumed to be maintained at very low levels in most cells due primarily to tight regulation in a negative feedback loop with MDM2.[31] Mutant p53 protein is often found at high levels in tumours as the pathway is abrogated by mutations.[32] The basal expression of p53 in BE cells is markedly higher than in MCF7 cells and would suggest a dysregulation of p53 control mechanisms with abrogation of the signalling downstream. The antibody used (DO-1) to detect p53 protein targets the transactivation domain, and we may expect any mutations to be outside this region.[33] Certainly, p53 missense alterations in the DNA binding domain are known to occur with high frequency in colorectal cancer.[34]

The form of the two distributions are very similar and for both cell lines we observe long tails. To help explain these, it has been shown that intrinsic DNA damage during the cell cycle of proliferating cells can trigger a response whereby p53 accumulates, but is not activated, in a pulsatile fashion.[35]. Due to the amplification effect throughout protein production, one would expect low average mRNA levels to result in a high-variance protein distribution, as small fluctuations in mRNA abundance get overly represented during translation. On the other hand, we expect a relatively high number of mRNA molecules to provide more robustness against such inevitable stochasticity and lead to a narrower protein distribution. Therefore, regardless of other downstream influences, we expected to see a direct effect of mRNA abundance on the variances of the distributions, as demonstrated in Fig 1B. One of the perhaps most striking features emerging from this analysis is that the coefficients of variations of the two cell lines’ distributions are so similar.

Another way to express this is that the fractional widths of the distributions, the ratio of width to mean, is almost identical despite variation in protein copy number. If the protein copy number were due purely to mRNA levels then variation in mRNA copy number should lead to variation in fractional width, but this is not observed. This suggests that basal p53 abundance is controlled by protein degradation, a hypothesis explored in greater detail below.

### Modelling suggests that basal p53 abundance is controlled by protein degradation

**Fig 3:**
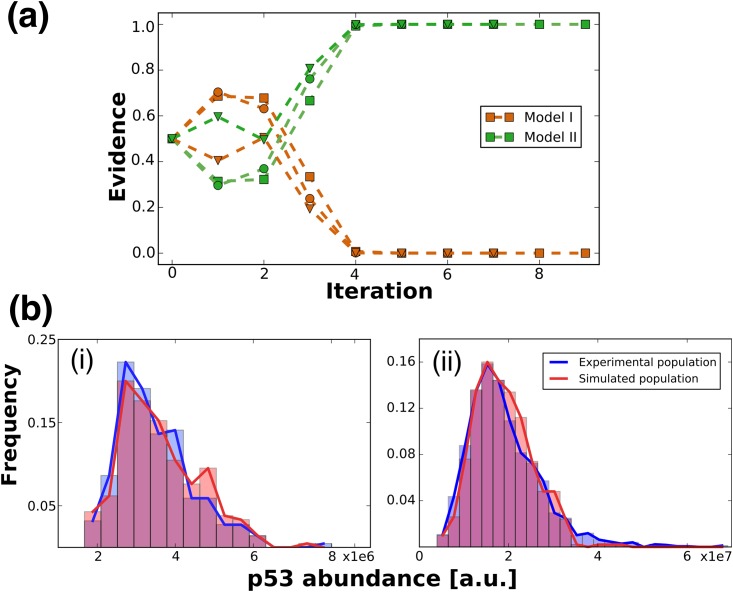
Results of computational analysis. **(A)** Result of iterative model selection algorithm. Evidence represents what ratio of accepted fits was obtained from a certain model. Dark orange and green curves show the transcription- (Model I) and the degradation-based (Model II) models, different markers denote replicates with different prior parameter distributions used at initialising the algorithm. **(B)** Comparison of experimental and simulated distributions. Histograms and solid curves outline the distribution of p53 abundance (measured as arbitrary fluorescence units) in (i) MCF7 and (ii) BE cells. Blue distributions were measured experimentally, red distributions were simulated during inference using the best fitting parameter combination. Note the difference in scale of p53 abundance in both histograms. *α* values of the fits: (i)*α* = 0.59, (ii) *α* = 0.34.

In Fig 1 we illustrate two models that could explain the observation of different p53 distributions in the two cell lines (see also Methods for details). We formalise our two competing models by introducing a scaling factor for the rate parameter of transcription (model I) or protein degradation (model II) and performed model selection. After the first few iterations (up to iteration 2, Fig 3A), the evidence for the degradation-based model quickly exceeds the transcription regulation model (beyond iteration 3, Fig 3A), with all the evidence supporting in the final parameter set model II. This result was consistent for a range of prior distributions for the model parameters (Fig 3A). The superiority of model I in the first section of iterations is due to the high acceptance rate that favours the more flexible transcription-based model; however, as the required quality of fit increases this model is not able to reproduce the experimental data well enough. Further datasets show a similar trend with all or at least a strong majority of the evidence excluding model I. (S1 Fig)

The coupled parameter inference concludes that p53 degradation is about 2.5 times slower in BE cells, consistent with our expectations given the general properties of the BE cell line (S2 Fig). An example population simulated with an instance of the final, best fitting group of parameter sets is shown in Fig 3B together with the experimentally measured data. The two distributions show no significant difference even at significance level of *α* = 0.3. The main difference between the distributions is in their tail: the simulated dataset failed to reproduce the cells lying far off from the main body of the distribution. However, as mentioned before, we assume that these significantly different cells are outliers arising from a not modelled, different population; therefore, less weight should be placed on these cells when evaluating the quality of a fit.

In addition, we confirmed the robustness of our result by using slightly modified versions of our basal expression models. As it is known that the dynamics of p53 are regulated through a negative feedback loop, the rate of protein degradation (k_3_) in our modified models is set to be a function of p53 abundance, k_3_*p53. We performed the same analysis on these models and arrived to the same conclusion (i.e. protein degradation model is selected unambiguously, S5 Fig); however, only poor fit could be achieved with these models. Furthermore, we also investigate additional models that can be considered in the framework of our base model. Details of the models and analysis results are included in supplementary material (S6 Fig). We find that a model with more free parameters has a slight advantage in model selection. Nevertheless, as the main aim of our study is to find the simplest mechanism that can explain the observed expression variance between cell lines, we conclude that protein degradation plays a significant role.

### Interpretations from transcriptional analysis

A system where protein-level effects dominate over transcriptional effects is thus supported by our results where we observe a higher coefficient of variation (CV) in the highly expressing BE cells as compared to MCF7 cells. We compared the variation in each distribution (S3 Fig) and the relative variability ranged from 1.5- to 2.4-fold higher variability in the BE cell distribution depending on whether outliers were excluded or included, respectively. Therefore, we propose that mRNA levels are on the same magnitude in both cell lines and regulation is protein-based.

**Fig 4:**
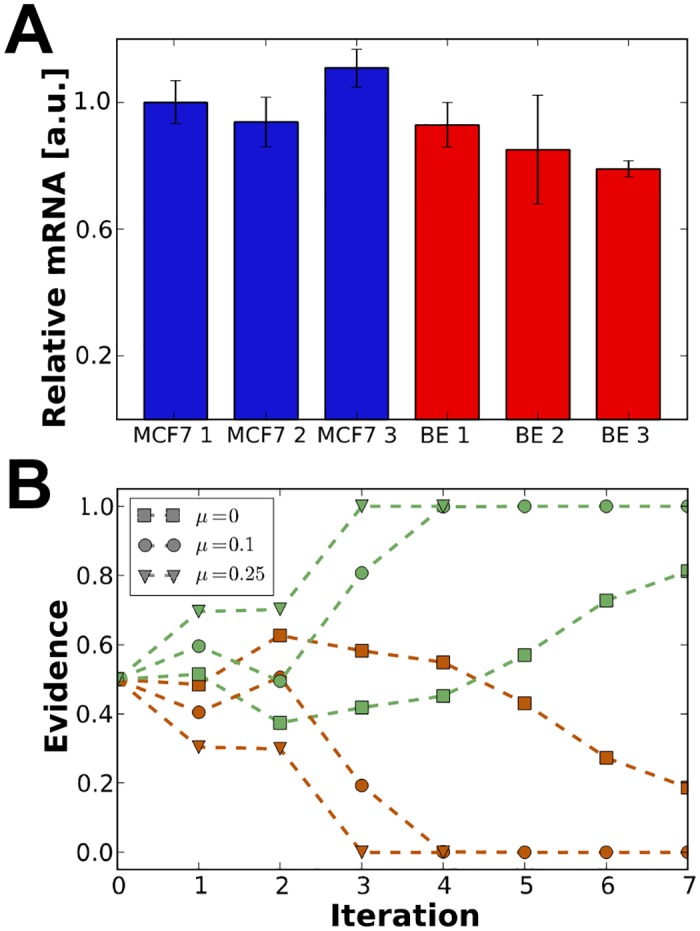
Results of transcriptional analysis and coupled model selection. **(A)** Relative mRNA levels measured by quantitative PCR. Bars represent relative mRNA yield, normalised to the first MCF7 sample. Error-bars show one standard deviation derived from the comparative C_T_ measurement. **(B)** Iterative model selection results incorporating qPCR information. Curves represent the evidence supporting model I (in dark orange) and model II (in green) for three weights of the term penalising differences in mRNA expression level. Graphs obtained with weight values 0, 0.1 and 0.25 are marked by squares, circles and triangles, respectively, as indicated in the legend.

To test this, quantitative PCR was performed using MCF7 and BE cell lines. Relative mRNA levels are calculated using the comparative C_T_ method and normalised to the first MCF7 sample (Fig 4A).[24] Despite a 2.6-fold higher average signal in the immunofluroescence data comparing protein levels, these results show that mRNA levels are on the same scale in both cell lines.

The results are incorporated into our simulation framework and we found that including a constraint on mRNA levels rules out the transcription-based model completely even for datasets where such absolute distinction was not possible previously. Fig 4B shows that for all non-zero values of the penalty weight, *μ* (see Materials and methods), determining the strength of the constraint, the final population did discriminate between the models (using e.g. the conventional scales for Bayesian model selection [36]), and the discrimination happened earlier in the iteration procedure as *μ* was increased.

In summary, it is clear that in these cell lines at least, basal levels of p53 are controlled by protein degradation rather than transcription.

## Conclusions

In this work we have shown that the combination of steady-state single cell measurements and computational techniques can be a powerful tool in understanding the mechanisms which help establish the baseline state of cells. We have measured the steady-state basal p53 mRNA and protein abundances in two human cancer cell lines. In particular, we found that, despite the high number of proteins under transcriptional control,[9] the regulation of basal p53 protein level in these human cancer cell lines is achieved through the rate of p53 protein degradation.

Understanding the contributions of transcriptional and translational control is important in ultimately understanding the underlying mechanism of cancer and how drugs may sometimes fail. For example, Zhang et al, reported that the variation in protein expression in colorectal tumours was poorly explained by the abundance in corresponding mRNA.[8] Certainly, a lack of correlation between mRNA and protein abundances has been increasingly reported as single cell analysis becomes increasingly quantitative. So far, system-wide studies have challenged one another in the conclusion that either transcriptional or translational control/variation contributes most to the variance in protein abundance.[4,7] As far as we are aware, this is the first report that concludes protein degradation is the predominant contributor to the variance in protein abundance of specifically basal p53 protein.

This makes sense in terms of the role p53 plays within the cell. Sometimes referred to as *the guardian of the genome*[37] p53 acts as an integrator of cellular stresses and must accumulate and respond rapidly. With this goal in mind, it stands to reason that it is perhaps faster to constitutively express and degrade p53 rather than activate the slow process of transcription and translation in times of stress. The data shown here does not imply that transcription rate and other processes has no effect on p53 abundance and dynamics. p53 mRNA itself might play an important role: indeed, it is known that p53 mRNA can directly bind to MDM2 and interrupt the protein-protein interaction that leads to p53 degradation.[38] An increase in MDM2 and p53 mRNA immunoprecipitation has been observed after treatment with the DNA damaging drug doxorubicin. Importantly there seems to be little correlation between transcript levels and corresponding protein abundance.

We chose to use a simple two-stage gene expression model to test in broad terms where the mechanisms of protein copy number regulation lie. Certainly, the model is extensible and may be developed to distinguish whether, for example, the variation in degradation rate is due to differences in MDM2 abundance or p53 phosphorylation status. Even though any such differences cannot be pinpointed with finer resolution using the model as presented here, the simplicity of the model is beneficial in guiding further study; altering individual reactions with sufficient accuracy and selectivity is pharmacologically challenging; however, disrupting general mechanisms is more achievable. Throughout the course of these iterative extensions our methods may be broadly applied to study other proteins of interest to help researchers further elucidate the mechanisms which control protein abundance and how these may be altered in disease or drug therapy.

## Supporting information

S1 Table Uniform prior distributions used for Bayesian analysis. Priors were used for the first iteration, in subsequent steps the previously derived posterior provided Results of Data I are presented in the main text, Data II and Data III in S1 and S2 Figs.
(PDF)

S2 Table Framework validation on synthetic datasets and parameter values used for synthetic data generation. To validate the discriminative ability of the applied methodology in its final form, we performed model selection on synthetic datasets, generated to reflect the properties of the actual measurements and our hypotheses. We use a basic model of one cell to simulate a population of MCF7 cells, consisted of nmcf7 = 150 observations. In order to show that our normalisation (i.e. expressing independent parameters by their ratios to the mRNA degradation rate) has no influence on the computation, the before-normalisation model is used, with parameter values as indicated in S2 Table. A BE cell data set of 300 cells is generated independently, with three different instances corresponding to cases when transcription, translation or protein degradation is regulated differently inside the cell to result in a higher baseline p53 expression level. Accordingly, the rate of transcription, translation or protein degradation, respectively, is chosen to be significantly different from that of MCF7 cells; while other values are perturbed slightly as we cannot expect the exact same values in a realistic system.
The synthesised data sets are used as target distributions in the model selection algorithm between Model I (transcription-based regulation)) and Model II (degradation-based control). In every other detail, the same procedure is used as described above for real observations.
All target data sets consist of the MCF7 data combined with one of the three synthetic BE measurements.
(PDF)

S1 Fig Results of computational analysis on the second experimental dataset. **(a)** Evidence supporting transcription (dark orange curves) and protein degradation (green curves) control. Different markers denote replicates with different prior parameter distributions used at initialising the algorithm. **(b)** Comparison of experimental and simulated distributions. Histograms and solid curves outline the distribution of p53 abundance (measured as arbitrary fluorescence units) in (i) MCF7 and (ii) BE cells. Experimentally measured distributions are in blue, simulated ones in red. *α* values of the fits are (i) 0.575, (ii) 0.51.
(PDF)

S2 Fig Parameter estimation results of the protein-degradation based model. Posterior distributions of the four parameters of Model II. Horizontal and vertical axes show possible parameter values and their probability, respectively. Prior distributions of the estimation algorithm were set to uniform ranges as summarised in “Data I—repeat III” in S1 Table.
(PDF)

S3 Fig Comparing relative widths of the distributions of basal p53 protein expression from MCF7 and BE cell lines. To compare the variation in the distributions we fit a gamma distribution to each data set. The shape (k) and scale (θ) parameters were 9.05 and 5.43 × 10^5^ for MCF7 cells and 2.44 and 5.19 × 10^6^ for BE cells, respectively. Consequently, the coefficient of variation (CV) of the MCF7 and BE gamma distribution fits were 0.33 and 0.64, respectively. The relative variability may be determined simply from the ratio of the CVs and ranges from 1.5- to 2.4-fold higher variability in the BE cell distribution depending on whether outliers are excluded or included, respectively.
(PDF)

S4 Fig Model selection results of three synthetic (validation) data sets. Evidence supporting transcription (dark orange curves) and protein degradation (green curves) control. Parameter values used for the generation of target datasets is as indicated in S2 Table.
(PDF)

S5 Fig Model selection results using modified (non-linear) models. Evidence supporting transcription (dark orange curves) and protein degradation (green curves) regulation. The models used in the inference and selection algorithm are identical to Model I and Model II with the exception of rate parameter of protein degradation changed from k_3_ to k_3_*p53, making the final protein degradation rate to k_3_*p53^2^.
(PDF)

S6 Fig Model selection results for four models. To explore further possibilities, we repeat our analysis with the inclusion of two more models, so a total of four models being compared by the model selection algorithm. The additional models are logical extensions of Model I and Model II: Model 0 corresponds to identical rates in all reactions in the two cell lines (i.e. s_1_ = s_2_ = 1) and Model III is the combination of I and II, when both transcription and protein degradation are allowed to differ between cell types. We compare these four models with settings identical to our previous analyses (priors are set according to Data set I—repeat 3 in S1 Table).
We find that, unsurprisingly, Model 0 and Model I get quickly discarded by the selection algorithm, unlike Model II and III, which confirms the importance of protein degradation in explaining the data (S6 Fig). It is also expected that Model III gains a higher level of evidence, as this model has an additional degree of freedom, giving it appropriate flexibility to match the data, especially the elongated tail of the distribution, better. Nevertheless, the ratio of evidences is not significant, so Model II cannot be discarded even in this case.
This result confirms that the true underlying dynamics are more complicated than pictured with our model, and we do not claim that protein degradation is the sole determinant in p53 expression levels. However, the importance of protein degradation is supported by our further analysis.
Figure shows evidence supporting expression control based on none of the rates (Model 0, blue line), transcription rate (Model I in dark orange), protein degradation rate (Model II, green) and transcription+protein degradation rates (Model III, purple).
(PDF)
