# Supplementary material for: Protein degradation rate is the dominant mechanism accounting for the differences in protein abundance of basal p53 in a human breast and colorectal cancer cell line
Source: PLoS One. 2017 May 10;12(5):e0177336. doi: 10.1371/journal.pone.0177336 (PMC5425217; doi:10.1371/journal.pone.0177336)
Supplement: S1 Table — Priors were used for the first iteration, in subsequent steps the previously derived posterior provided Results of Data I are presented in the main text, Data II and Data III in S1 and S2 Figs. (PDF) [file pone.0177336.s001.pdf]

	k_1	k_2	k_3	s_1	s_2
Data I – repeat 1	[1, 20]	[5e5, 1.2e7]	[0, 22]	[0, 6]	[0, 2]
Data I – repeat 2	[1, 20]	[5e5, 1.2e7]	[0, 35]	[0, 6]	[0, 2]
Data I – repeat 3	[1, 10]	[2e6, 1.2e7]	[0, 28]	[0, 6]	[0, 2]
Data II – repeat 1	[0, 18]	[5e5, 1e7]	[0, 15]	[0, 4]	[0, 2]
Data II – repeat 2	[0, 15]	[1e6, 1e7]	[0, 12]	[0, 4]	[0, 2]
Data II – repeat 3	[0, 10]	[2e6, 1e7]	[0, 15]	[0, 4]	[0, 2]
