# Supplementary material for: Protein degradation rate is the dominant mechanism accounting for the differences in protein abundance of basal p53 in a human breast and colorectal cancer cell line
Source: PLoS One. 2017 May 10;12(5):e0177336. doi: 10.1371/journal.pone.0177336 (PMC5425217; doi:10.1371/journal.pone.0177336)
Supplement: S2 Table — To validate the discriminative ability of the applied methodology in its final form, we performed model selection on synthetic datasets, generated to reflect the properties of the actual measurements and our hypotheses. We use a basic model of one cell to simulate a population of MCF7 cells, consisted of nmcf7 = 150 observations. In order to show that our normalisation (i.e. expressing independent parameters by their ratios to the mRNA degradation rate) has no influence on the computation, the before-normalisation model is used, with parameter values as indicated in S2 Table. A BE cell data set of 300 cells is generated independently, with three different instances corresponding to cases when transcription, translation or protein degradation is regulated differently inside the cell to result in a higher baseline p53 expression level. Accordingly, the rate of transcription, translation or protein degradation, respectively, is chosen to be significantly different from that of MCF7 cells; while other values are perturbed slightly as we cannot expect the exact same values in a realistic system. The synthesised data sets are used as target distributions in the model selection algorithm between Model I (transcription-based regulation)) and Model II (degradation-based control). In every other detail, the same procedure is used as described above for real observations. All target data sets consist of the MCF7 data combined with one of the three synthetic BE measurements. (PDF) [file pone.0177336.s002.pdf]

	k_1	k_2	k_3	k_{mrna_deg}
MCF7	30	5e6	30	5
BE - transcription	248	5.3e6	28	4.8
BE - translation	31	1.6e7	28	4.8
BE – protein degradation	31	5.3e6	5.6	4.8
