# Supplementary figures and images for: Protein degradation rate is the dominant mechanism accounting for the differences in protein abundance of basal p53 in a human breast and colorectal cancer cell line
Source: PLoS One. 2017 May 10;12(5):e0177336. doi: 10.1371/journal.pone.0177336 (PMC5425217; doi:10.1371/journal.pone.0177336)

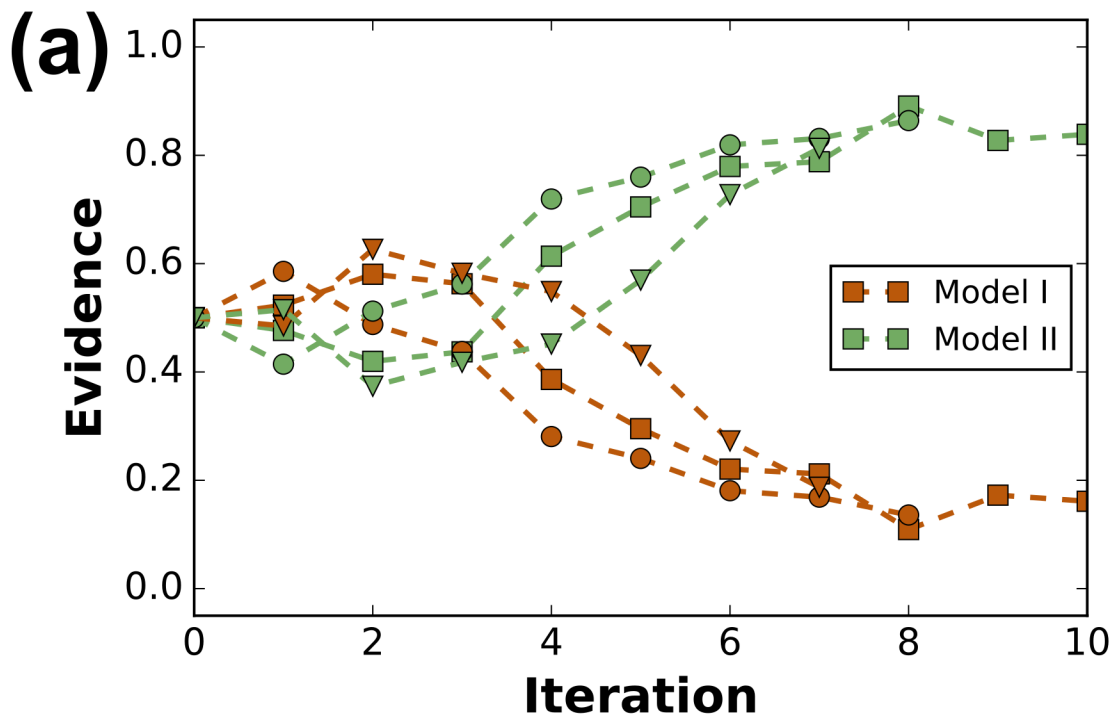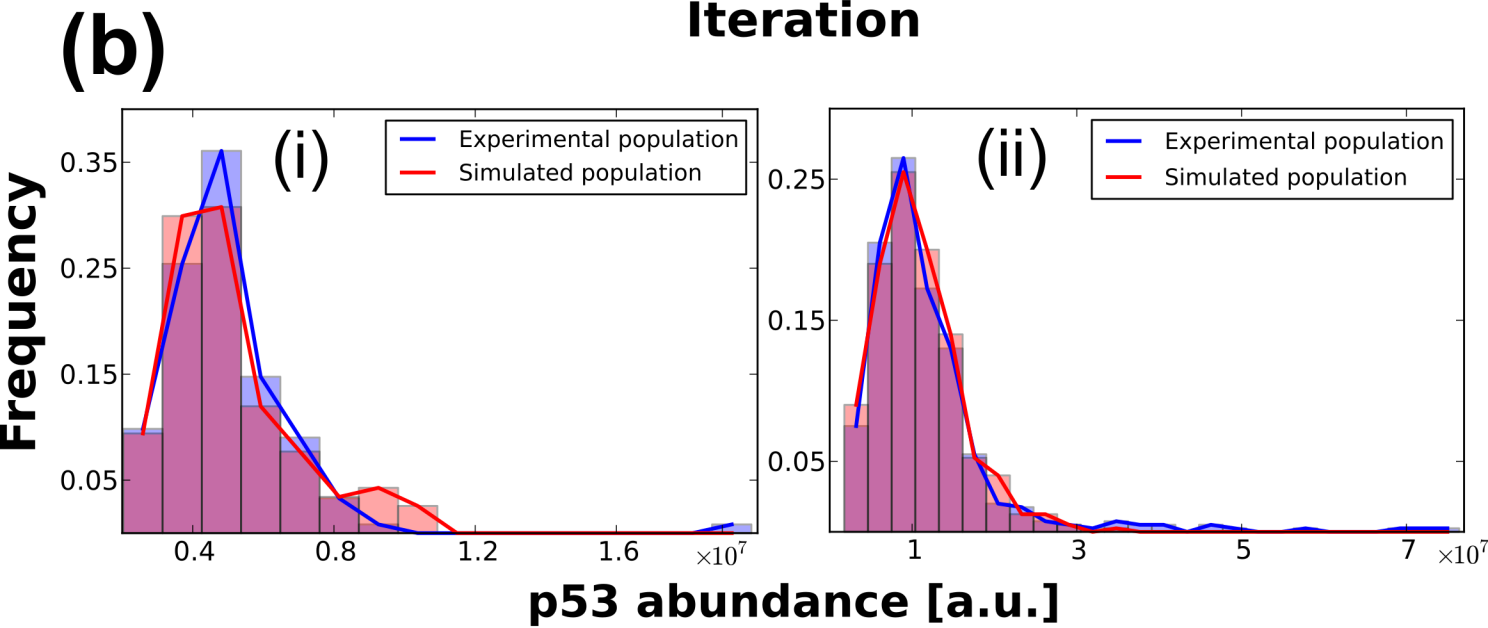

Supplement: S1 Fig — (a) Evidence supporting transcription (dark orange curves) and protein degradation (green curves) control. Different markers denote replicates with different prior parameter distributions used at initialising the algorithm. (b) Comparison of experimental and simulated distributions. Histograms and solid curves outline the distribution of p53 abundance (measured as arbitrary fluorescence units) in (i) MCF7 and (ii) BE cells. Experimentally measured distributions are in blue, simulated ones in red. α values of the fits are (i) 0.575, (ii) 0.51. (PDF) [file pone.0177336.s003.pdf]

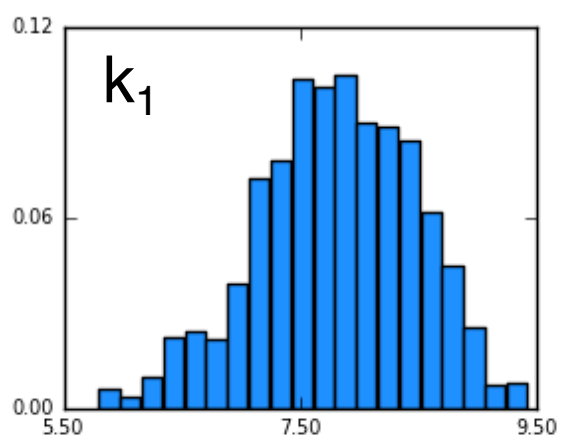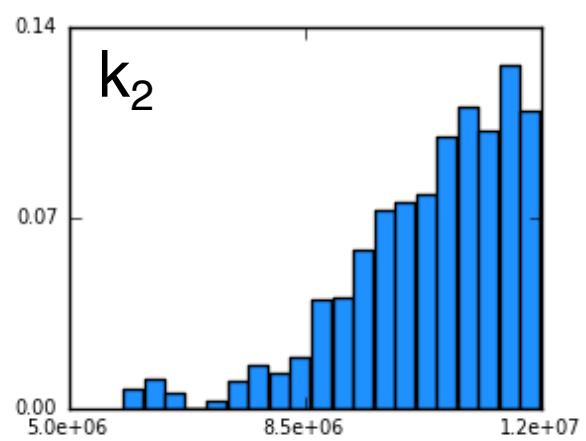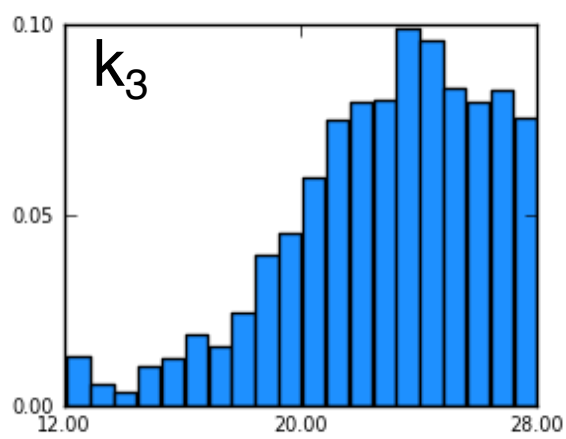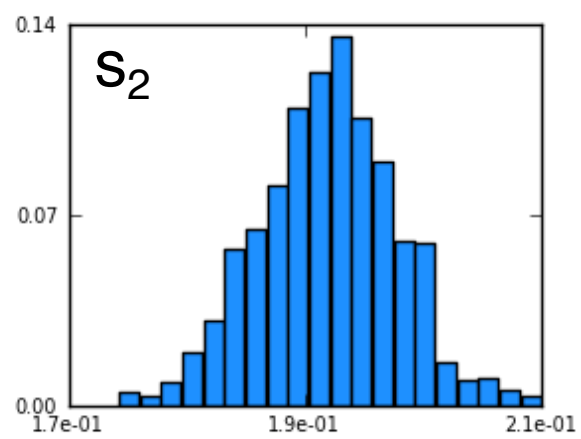

Supplement: S2 Fig — Posterior distributions of the four parameters of Model II. Horizontal and vertical axes show possible parameter values and their probability, respectively. Prior distributions of the estimation algorithm were set to uniform ranges as summarised in “Data I—repeat III” in S1 Table. (PDF) [file pone.0177336.s004.pdf]

i) MCF7 ii)

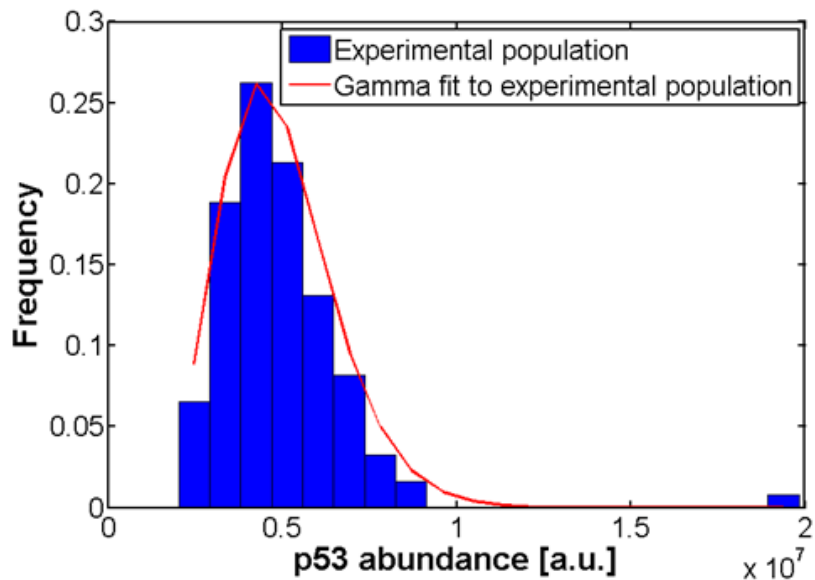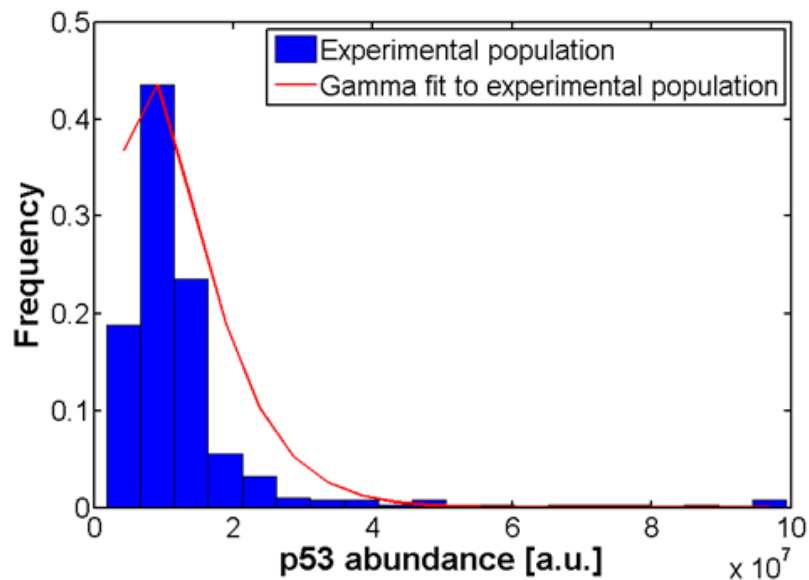

Supplement: S3 Fig — To compare the variation in the distributions we fit a gamma distribution to each data set. The shape (k) and scale (θ) parameters were 9.05 and 5.43 × 10^5^ for MCF7 cells and 2.44 and 5.19 × 10^6^ for BE cells, respectively. Consequently, the coefficient of variation (CV) of the MCF7 and BE gamma distribution fits were 0.33 and 0.64, respectively. The relative variability may be determined simply from the ratio of the CVs and ranges from 1.5- to 2.4-fold higher variability in the BE cell distribution depending on whether outliers are excluded or included, respectively. (PDF) [file pone.0177336.s005.pdf]

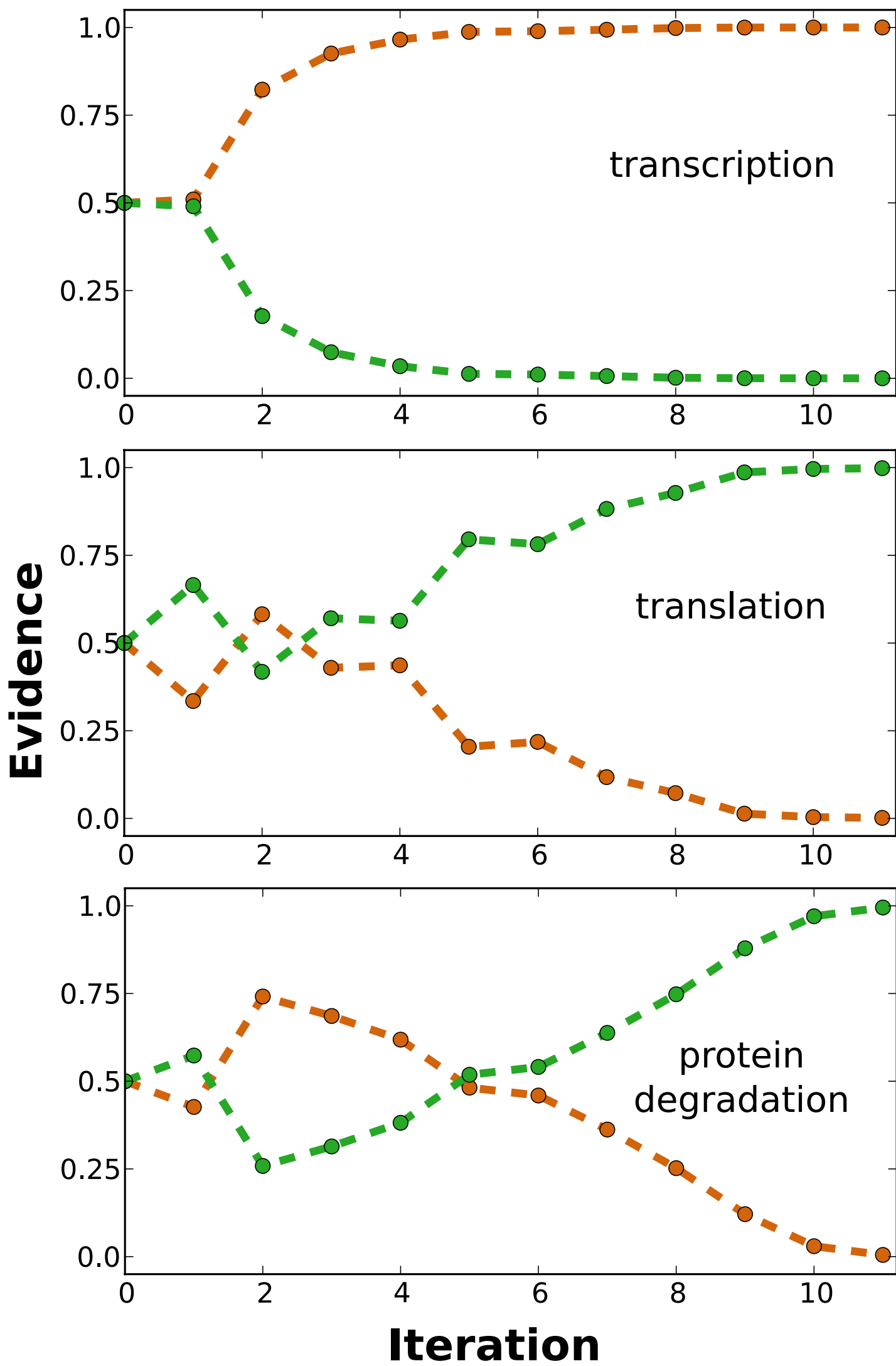

Supplement: S4 Fig — Evidence supporting transcription (dark orange curves) and protein degradation (green curves) control. Parameter values used for the generation of target datasets is as indicated in S2 Table. (PDF) [file pone.0177336.s006.pdf]

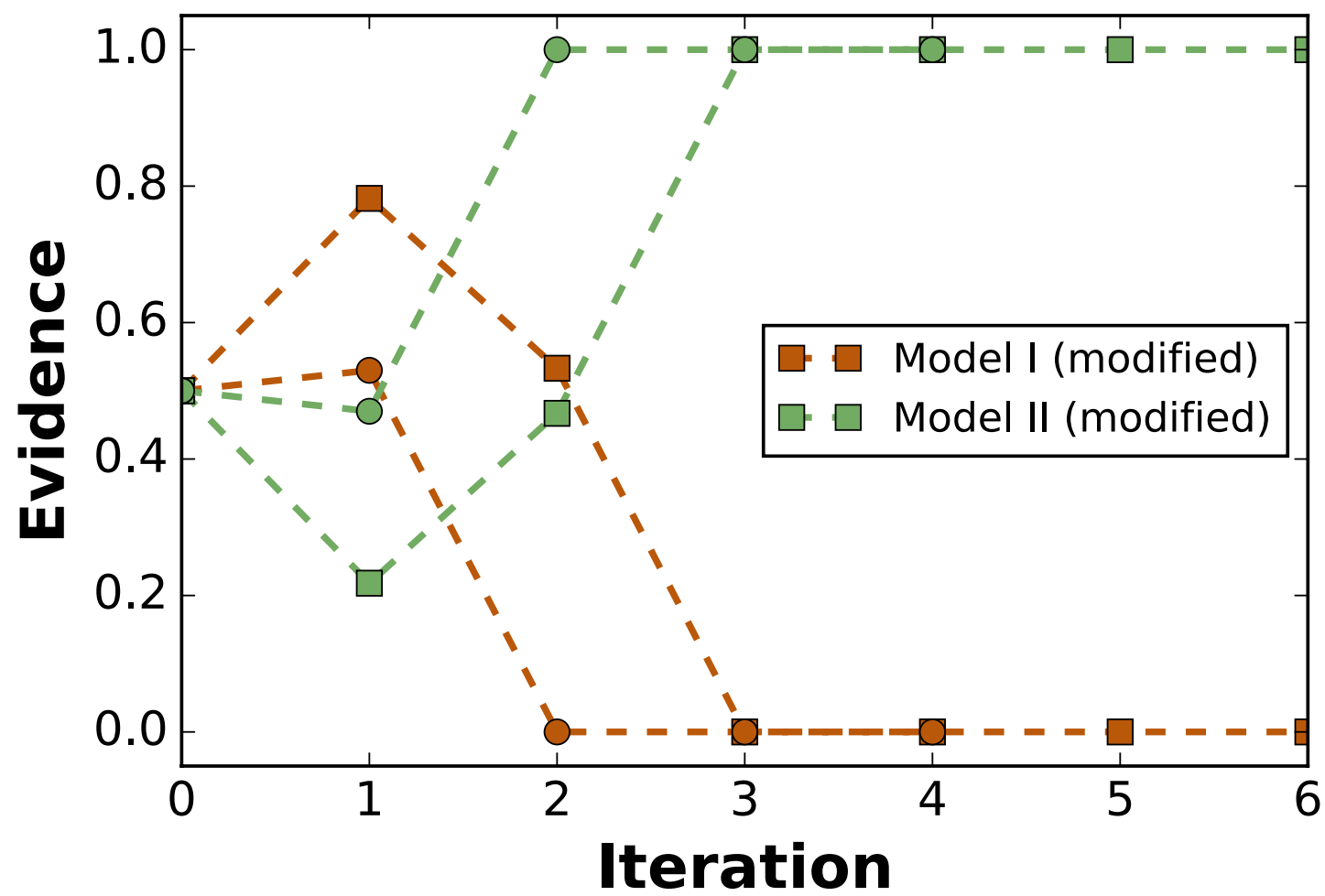

Supplement: S5 Fig — Evidence supporting transcription (dark orange curves) and protein degradation (green curves) regulation. The models used in the inference and selection algorithm are identical to Model I and Model II with the exception of rate parameter of protein degradation changed from k3 to k3*p53, making the final protein degradation rate to k3*p532. (PDF) [file pone.0177336.s007.pdf]

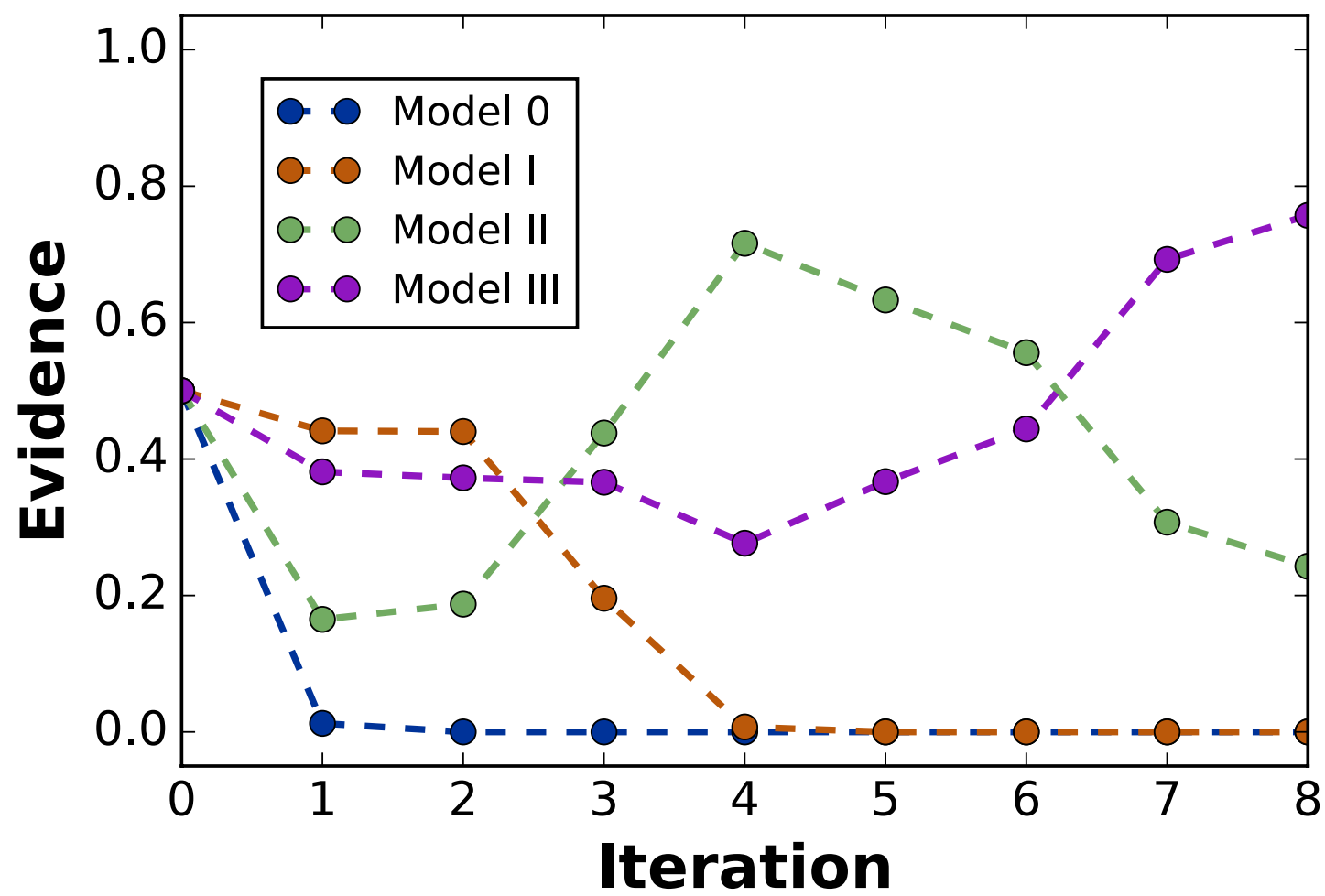

Supplement: S6 Fig — To explore further possibilities, we repeat our analysis with the inclusion of two more models, so a total of four models being compared by the model selection algorithm. The additional models are logical extensions of Model I and Model II: Model 0 corresponds to identical rates in all reactions in the two cell lines (i.e. s1 = s2 = 1) and Model III is the combination of I and II, when both transcription and protein degradation are allowed to differ between cell types. We compare these four models with settings identical to our previous analyses (priors are set according to Data set I—repeat 3 in S1 Table). We find that, unsurprisingly, Model 0 and Model I get quickly discarded by the selection algorithm, unlike Model II and III, which confirms the importance of protein degradation in explaining the data (S6 Fig). It is also expected that Model III gains a higher level of evidence, as this model has an additional degree of freedom, giving it appropriate flexibility to match the data, especially the elongated tail of the distribution, better. Nevertheless, the ratio of evidences is not significant, so Model II cannot be discarded even in this case. This result confirms that the true underlying dynamics are more complicated than pictured with our model, and we do not claim that protein degradation is the sole determinant in p53 expression levels. However, the importance of protein degradation is supported by our further analysis. Figure shows evidence supporting expression control based on none of the rates (Model 0, blue line), transcription rate (Model I in dark orange), protein degradation rate (Model II, green) and transcription+protein degradation rates (Model III, purple). (PDF) [file pone.0177336.s008.pdf]
